# Identification and Characterization of the Pyruvate Dehydrogenase E1 Gene in the Oriental River Prawn, *Macrobrachium nipponense*

**DOI:** 10.3389/fendo.2021.752501

**Published:** 2021-11-01

**Authors:** Shubo Jin, Yuning Hu, Hongtuo Fu, Sufei Jiang, Yiwei Xiong, Hui Qiao, Wenyi Zhang, Yongsheng Gong, Yan Wu

**Affiliations:** ^1^ Key Laboratory of Freshwater Fisheries and Germplasm Resources Utilization, Ministry of Agriculture, Freshwater Fisheries Research Center, Chinese Academy of Fishery Sciences, Wuxi, China; ^2^ Wuxi Fisheries College, Nanjing Agricultural University, Wuxi, China

**Keywords:** *Macrobrachium nipponense*, *PDHE1*, qPCR analysis, RNAi, male sexual development

## Abstract

Pyruvate dehydrogenase E1 (*PDHE1*) is thought to play essential roles in energy metabolism, and a previous study suggested that it also has potential regulatory roles in male sexual development in the oriental river prawn, *Macrobrachium nipponense*. In this study, we used rapid amplification of cDNA ends, quantitative polymerase chain reaction (qPCR), *in situ* hybridization, western blotting, RNA interference (RNAi), and histological analyses to assess the potential functions of *Mn-PDHE1* in the sexual development of male *M. nipponense*. The full cDNA sequence of *Mn-PDHE1* was 1,614 base pairs long, including a 1,077 base pair open reading frame that encodes 358 amino acids. qPCR analysis revealed the regulatory functions of *PDHE1* in male sexual development in *M*. *nipponense* and in the metamorphosis process. *In situ* hybridization and western blot results indicated that *PDHE1* was involved in testis development, and RNAi analysis showed that *PDHE1* positively regulated the expression of insulin-like androgenic gland factor in *M. nipponense*. Compared with the cell types in the testes of control prawns, histological analysis showed that the number of sperm was dramatically lower after test subjects were injected with *Mn-PDHE1* dsRNA, whereas the numbers of spermatogonia and spermatocytes were higher. Sperm constituted only 1% of cells at 14 days after injection in the RNAi group. This indicated that knockdown of the expression of *PDHE1* delayed testis development. Thus, *PDHE1* has positive effects on male sexual development in *M*. *nipponense*. This study highlights the functions of P*DHE1* in *M*. *nipponense* and its essential roles in the regulation of testis development.

## Introduction

The oriental river prawn, *Macrobrachium nipponense* (Crustacea; Decapoda; Palaemonidae), is widely distributed in China and other Asian countries, and had an annual aquaculture production of 205,010 tons in 2016 (1–4). Male prawns show better growth performance than their female counterparts, as they grow faster and reach larger size by harvest time (2). However, the rapid development of the testis during the reproductive season restricts the sustainable development of *M*. *nipponense* aquaculture. Jin et al. (5) reported that the testis of *M*. *nipponense* can reach sexual maturity within 40 days after hatching, thus inbreeding can occur between newly hatched prawns. This will lead to small-sized prawns, degradation of germplasm resources, and decreased ability of offspring to tolerate adverse conditions. A better understanding of male sexual differentiation and development mechanisms is urgently needed to develop a technique to produce all male progeny on a commercial scale and to regulate the process of testis development in *M*. *nipponense*.

The androgenic gland and its secreted hormones play important positive regulatory roles in the process of male sexual differentiation and development in crustacean species, and it is especially true for testis development (6, 7). The testis also has significant effects on male sexual differentiation, sexual maturity, and reproductive capability in crustacean species (8–11). Jin et al. (5) previously showed that the androgenic gland regulates testis development in *M*. *nipponense*.

Many environmental factors, including temperature, illumination, and the presence of chemical pollutants, can regulate sexual differentiation and development by affecting the expression profiles of sex-related genes (12). Jin et al. (13) previously described significant morphological differences in the testis and androgenic gland of *M*. *nipponense* between the reproductive and non-reproductive seasons. Using transcriptome profiling to analyse the testis and androgenic gland during the two seasons, it was found that glycolysis/gluconeogenesis and the tricarboxylic acid (TCA) cycle may play essential roles in promoting the process of male sexual differentiation and development in *M*. *nipponense* by providing ATP. Pyruvate dehydrogenase E1 (*PDHE1*) is an important gene that is enriched in both the glycolysis/gluconeogenesis and TCA metabolic pathways (13). The PDH complex (*PDHc*) plays essential roles in the glycolysis pathway. Glucose is reduced to form pyruvate through glycolysis. *PDHc* can catalyse the oxidative decarboxylation of pyruvate to become acetyl-CoA, which is required by the TCA cycle, depending on the needs of cells (14, 15). PDH deficiency means that pyruvate cannot be converted into acetyl-CoA but instead is reduced to lactic acid, thus affecting the TCA cycle and resulting in decreased ATP production (16). An abnormal TCA cycle leads to metabolic disorders and tissue damage. Therefore, *PDHc* plays an important role in maintaining the normal metabolism of animals. *PDHE1* is the key enzyme component of *PDHc*, as it catalyses the rate-limiting step of the oxidative decarboxylation of pyruvate (17, 18).

The goal of this study was to verify the important functions of *PDHE1* in the process of male sexual development in *M. nipponense* using rapid amplification of cDNA ends (RACE) cloning, quantitative polymerase chain reaction (qPCR), *in situ* hybridization, western blot, RNA interference (RNAi), and histological analyses. We focused on the potential regulatory roles of *PDHE1* on male sexual development in *M. nipponense*. Results of this study provide essential data that can be used to establish a technique to regulate testis development in *M. nipponense*.

## Materials and Methods

### Ethics Statement

All experiments involving *M*. *nipponense* were approved by the Institutional Animal Care and Use Ethics Committee of the Freshwater Fisheries Research Center, Chinese Academy of Fishery Sciences (Wuxi, China).

### Sample Preparation

Healthy adult male and female *M. nipponense* with the body weight of 2.87-4.51 g for male prawns and the body weight of 2.37-3.45 g for female prawns were obtained from Tai Lake in Wuxi, China (120°13’44”E, 31°28’22”N). These specimens were transferred to a 500 L tank in the lab and maintained in aerated freshwater at room temperature (28°C) with a dissolved oxygen content ≥ 6 mg/L. The specimens were maintained under lab conditions for 72 h prior to tissue collection. The testis, ovary, hepatopancreas, muscle, eyestalk, gill, heart, and brain were collected from five different prawns (19). The ovarian sample was the mixed sample of the whole ovarian reproductive cycle, which included ovarian stage I (O I), O II, O III, O IV, O V. The five standard phases of the ovarian reproductive cycle have been well described by previous study (20). Additionally, one male prawn and one female prawn were mated and young were hatched in the lab to produce the full-sib population. Specimens for the different developmental stages were collected from this full-sib population during their maturation process (19). For males, the testis and androgenic gland were collected from five specimens during the reproductive season (at 28°C in summer) and from five specimens during the non-reproductive season (at 15°C in winter). Five prawns were also sampled from each of the five standard phases of the ovarian reproductive cycle. The samples were treated with phosphate buffered saline and immediately frozen in liquid nitrogen until used for RNA and protein extraction to prevent total RNA and protein degradation.

### RACE

We followed previously described procedures to conduct RACE cloning (21, 22). The specific primers used for *Mn-PDHE1* RACE cloning were listed in **Table 1**, designed by the Primer-BLAST tool in NCBI (http://www.ncbi.nlm.nih.gov/tools/primer-blast/) (**Table 1**). **Table 2** lists sequences of *PDHE1* from different species. The phylogenetic tree was constructed by MEGA X using the maximum-likelihood method with 1000 bootstrap replications.

**Table 1:** Universal and specific primers used in this study.

Primer name	Nucleotide Sequence (5′→3′)	Purpose
PDHE1-3GSP1	CTGAGATCTGTGCTAGAATAG	FWD first primer for PDHE1 3′ RACE
PDHE1-3GSP2	CCCAGTGATTCGTGTAACGGG	FWD second primer for PDHE1 3′ RACE
PDHE1 -5GSP1	TCAGGAACACCCCTTCATCCC	RVS first primer for PDHE1 5′ RACE
PDHE1 -5GSP2	TACTCTTGTTGTGGAGAAACT	RVS second primer for PDHE1 5′ RACE
3′RACE OUT	TACCGTCGTTCCACTAGTGATTT	RVS first primer for 3′ RACE
3′RACE IN	CGCGGATCCTCCACTAGTGATTTCACTATAGG	RVS second primer for 3′ RACE
5′RACE OUT	CATGGCTACATGCTGACAGCCTA	FWD first primer for 5′ RACE
5′RACE IN	CGCGGATCCACAGCCTACTGATGATCAGTCGATG	FWD second primer for 5′ RACE
PDHE1 -RTF	TGACCTTAACGGCAACGAGG	FWD primer for PDHE1 expression
PDHE1 -RTR	TCCAGGGCAGAATTGAGAGC	RVS primer for PDHE1 expression
IAG-RTF	CGCCTCCGTCTGCCTGAGATAC	FWD primer for IAG expression
IAG-RTR	CCTCCTCCTCCACCTTCAATGC	RVS primer for IAG expression
EIF-F	CATGGATGTACCTGTGGTGAAAC	FWD primer for EIF expression
EIF-R	CTGTCAGCAGAAGGTCCTCATTA	RVS primer for EIF expression
PDHE1 anti-sense Probe	GTTGACCTGCTGCTACTCTTGTTGTGGAGAAACTACGG	Probe for PDHE1 ISH analysis
PDHE1 sense Probe	CCGTAGTTTCTCCACAACAAGAGTAGCAGCAGGTCAAC	Probe for PDHE1 ISH analysis
PDHE1 RNAi-F	TAATACGACTCACTATAGGGGTGCTCTTAGCACTGGAGGC	FWD primer for RNAi analysis
PDHE1 RNAi-R	TAATACGACTCACTATAGGGCCAAGTAGTGGAAGGCAGGA	RVS primer for RNAi analysis

**Table 2:** Species used for the construction of PDHE1 phylogenetic tree.

Species	Accession number
*Macrobrachium nipponense*	MW366892
*Penaeus vannamei*	ROT67345.1
*Blattella germanica*	PSN33167.1
*Zootermopsis nevadensis*	XP_021917776.1
*Brunneria borealis*	QBH74078.1
*Orthoderella ornate*	QBH74091.1
*Nicoletia phytophila*	QBH74090.1
*Thermobia domestica*	QBH74092.1
*Atta cephalotes*	XP_012055795.1
*Atta colombica*	XP_018049360.1
*Bemisia tabaci*	XP_018907986.1
*Aedes albopictus*	Aedes albopictus XP

### qPCR Analysis

The relative mRNA expression levels of *Mn-PDHE1* in the different tissues were measured using qPCR, which was performed on the Bio-Rad iCycler iQ5 Real-Time PCR System (Bio-Rad, Hercules, CA, USA) and used to carry out the SYBR Green RT-qPCR assay (21, 22). **Table 1** lists the primers used for qPCR analysis. The expression levels between different tissues were measured by using 2^-ΔΔCT^ method (23).

### *In Situ* Hybridization

*In situ* hybridization (22, 24) was performed to analyse the mRNA locations of Mn-PDHE1. Primer5 software was used to design the anti-sense and sense probes for chromogenic *in-situ* hybridization, with the digoxygenin signal based on the cDNA sequence of *Mn-PDHE1* (**Table 1**). The primers for *in situ* hybridization analysis were synthesized by Shanghai Sangon Biotech Company (Shanghai, China). Slides were examined under light microscope for evaluation the cell types.

### Western Blot Analysis

Western blot analysis was conducted on the 20 mg tissues samples following the previous study (25). The total protein concentrations were quantified using the Bradford method (26). A total of 50 mg protein from each sample was separated on a 10% sodium dodecyl-polyacrylamide gel. The gel was then transferred to a polyvinylidene fluoride membrane (Millipore, Bedford, MA, USA).

### RNAi Analysis

RNAi was performed to analyse the potential regulatory roles of *Mn-PDHE1* in *M*. *nipponense*. For this analysis, 300 healthy mature male *M*. *nipponense* were collected (body weight 3.34–4.47 g). These prawns were randomly divided into the RNAi group and the control group, each containing 150 male prawns. Each group was further divided into three replicates of 50 prawns, which were maintained in 500 L tanks at 28°C with dissolved oxygen content ≥ 6 mg/L. The procedures for designing specific RNAi primer and synthesizing the dsRNA have been well described in previous studies (24, 27). The concentration of *Mn-PDHE1* dsRNA was measured using a spectrophotometer and diluted to 4 μg/μl. The prawns in the RNAi group were injected with 4 μg/g of *Mn-PDHE1* dsRNA (24, 27) and those in the control group were injected with an equal volume of green fluorescent protein, based on their body weights. qPCR was used to investigate the *PDHE1* mRNA expression in the testis after injection (N=5). To assess the regulatory relationship between *Mn-PDHE1* and insulin-like androgenic gland hormone (*Mn-IAG*), the *IAG* mRNA expression levels were also measured in the same *Mn-PDHE1* dsRNA-treated prawns on the same days (N=10).

### Histological Observations

Morphological differences between the testis of prawns in the RNAi group and the testis of samples in the control group were observed using haematoxylin and eosin (HE) staining after *Mn-PDHE1* dsRNA injection (N = 5). We followed the methods described in previous studies (28, 29). The prepared testis samples were observed using an Olympus SZX16 microscope (Olympus Corporation, Tokyo, Japan).

### Statistical Analysis

SPSS Statistics 23.0 (IBM, Armonk, NY, USA) was used to conduct all statistical analyses. Statistical differences were identified by one-way analysis of variance followed by least significant difference and Duncan’s multiple range tests. The statistical difference between the control group and the RNAi group on the same day was assessed using the paired t-test. Quantitative data were expressed as mean ± standard deviation. A *p*-value < 0.05 was considered to be statistically significant.

## Results

### Genome and cDNA Sequence Analysis

**Figure 1:**
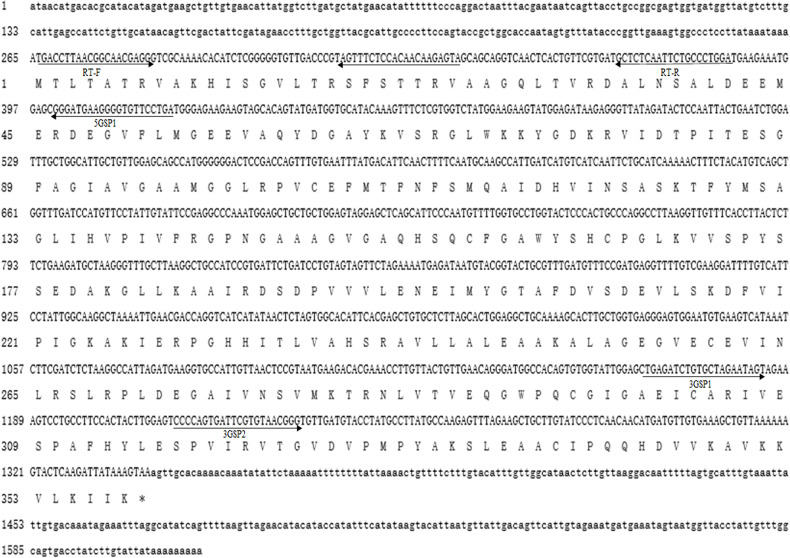
Nucleotide and deduced amino acid sequence of *Mn-PDHE1*. The nucleotide sequence is displayed in the 5′–3′ directions and numbered at the left. The deduced amino acid sequence is shown in a single capital letter amino acid code. 3′ UTR and 5′ UTR are shown with lowercase letters. Codons are numbered at the left with the methionine (ATG) initiation codon, an asterisk denotes the termination codon (TGA).

The full cDNA sequence of Mn-PDHE1 was 1,614 base pairs (bp) long, with a 5′ untranslated region (UTRs) and a 3′ UTR of 264 bp and 273 bp, respectively (**Figure 1**). *Mn-PDHE1* included a 1,077 bp open reading frame that encoded 358 amino acids. The sequence was submitted to NCBI with accession number MW366892. The molecular weight of the *Mn-PDHE1* protein was 38.539 kDa, and the theoretical isoelectric point of *Mn-PDHE1* protein was 6.56.

**Figure 2:**
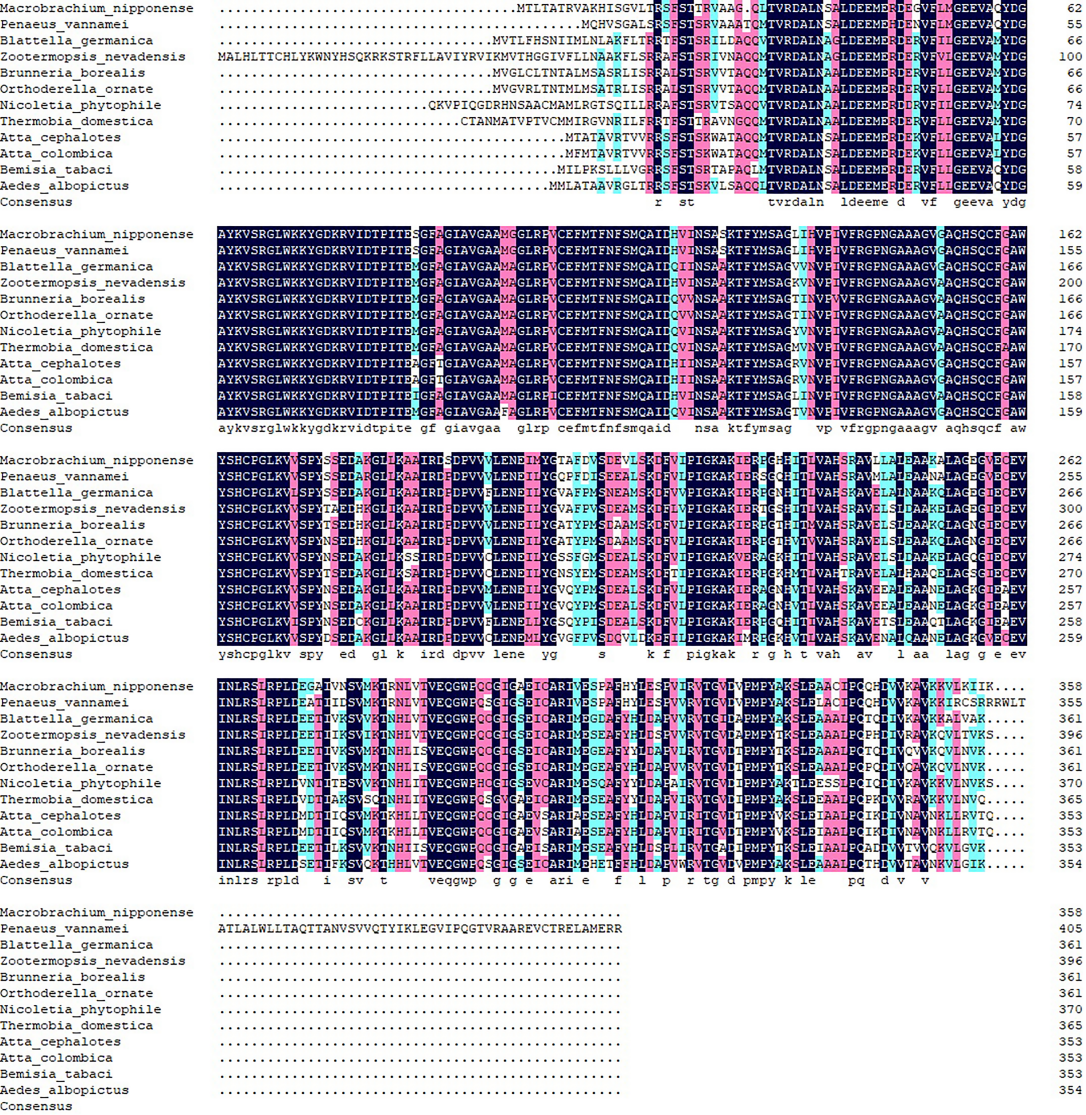
The alignment of amino acid sequences of *PDHE1* from different species.

**Figure 3:**
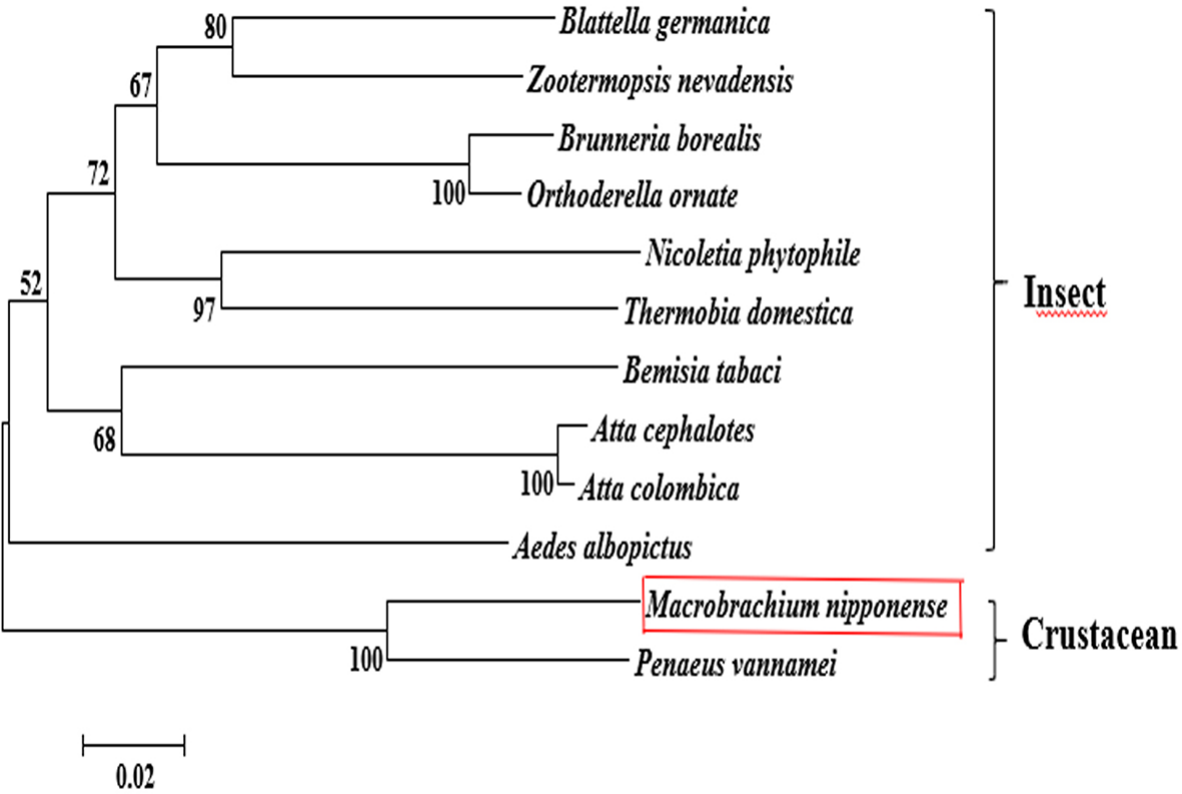
The phylogenetic tree of *PDHE1* from different organisms based on amino acid sequence comparisons. Species names and types of *PDHE1* are listed on the right of the tree. Red rectangles indicated *M. nipponense*.

The BLASTP similarity analysis in NCBI showed that *Mn-PDHE1* had the highest sequence similarity with that of *Penaeus vannamei* (90.72%). The similarities with other *PDHE1* sequences from insect species were also > 70%, including *Blattella germanica* (81.66%), *Zootermopsis nevadensis* (81.47%), *Brunneria borealis* (77.31%), and *Nicoletia phytophila* (79.30%) (**Figure 2**). The amino acid sequences listed in **Table 2** were used to construct the phylogenetic tree, which showed two main clusters. The amino acid sequence of *Mn-PDHE1* clustered with that of *P. vannamei*, whereas the amino acid sequences of *PDHE1* from the insect species clustered together as another group. Thus, *Mn-PDHE1* has the closest evolutionary relationship with that of *P. vannamei* and a dramatically distant evolutionary relationship with those from insect species (**Figure 3**).

### Expression of *Mn-PDHE1* in Different Tissues and Developmental Stages

**Figure 4:**
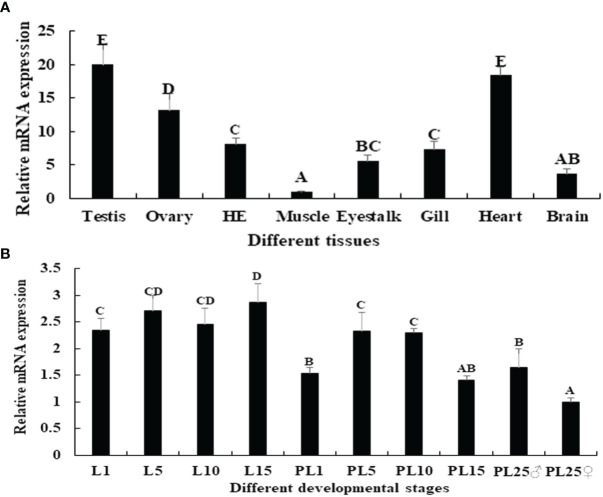
**(A)** Expression characterization *of Mn-PDHE1* in different tissues. **(B)** Expression characterization *of Mn-PDHE1* in different developmental stages. The amount of Mn-PDHE1 mRNA was normalized to the *EIF* transcript level. Data are shown as mean ± SD (standard deviation) of tissues from three separate individuals. Capital letters indicate expression difference between different samples.

Among the tissues tested, the *Mn-PDHE1* mRNA expression level was highest in the testis, followed by the heart and ovary, which showed significant difference with other tested tissues (*p* < 0.05). The lowest expression was observed in muscle, and the expression levels in the testis and ovary were 20.37-fold and 13.18-fold higher than that in muscle, respectively (**Figure 4A**).

Juvenile prawns can be visually distinguished as male or female for the first time at post-larval developmental stage 25 (PL25) by observing the presence of external gonadal features. Overall, the expression levels of *Mn-PDHE1* were higher in the larval developmental stages compared with the post-larval developmental stages. During larval and post-larval development, the highest expression level of *Mn-PDHE1* was observed in larval developmental stage 15 (L15), whereas the lowest expression level was observed in PL25 females (PL25♀). The expression at L15 was 2.87-fold higher than that of PL25♀. During post-larval development, expression peaked at PL5 and then gradually decreased to PL15. The expression level in PL25 males (PL25♂) was 1.64-fold higher and significantly different than that of PL25♀ (*p* < 0.05) (**Figure 4B**).

### Expression of *Mn-PDHE1* in Different Ovarian Developmental Stages

**Figure 5:**
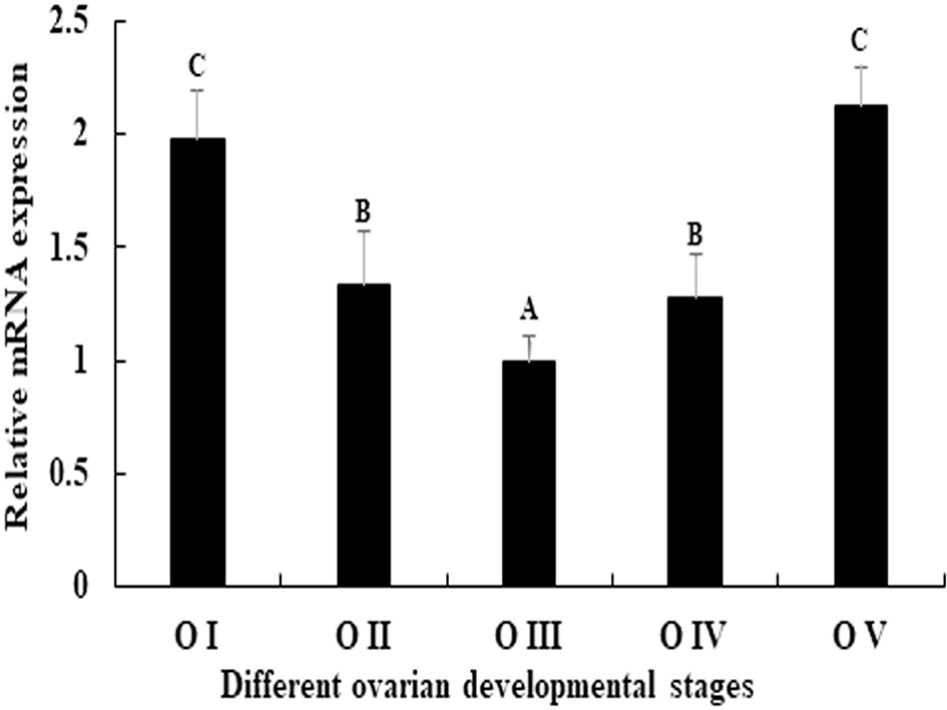
Expression characterization of Mn-PDHE1 in different reproductive cycles of ovary. The amount of Mn-PDHE1 mRNA was normalized to the EIF transcript level. Data are shown as mean ±SD (standard deviation) of tissues from three separate individuals. Capital letters indicate expression difference between different samples.

The expression pattern of *Mn-PDHE1* was analysed in different ovarian developmental cycle of *M*. *nipponense*. The expression of *Mn-PDHE1* reached the bottom at O III during the ovarian developmental cycle, and showed significant difference with other tested stages (*p* < 0.05). The highest expression level was observed in O V, followed by O I, which is 2.13-folder and 1.98-folder higher than that of O III, respectively (**Figure 5**).

### Western Blot Analysis

**Figure 6:**
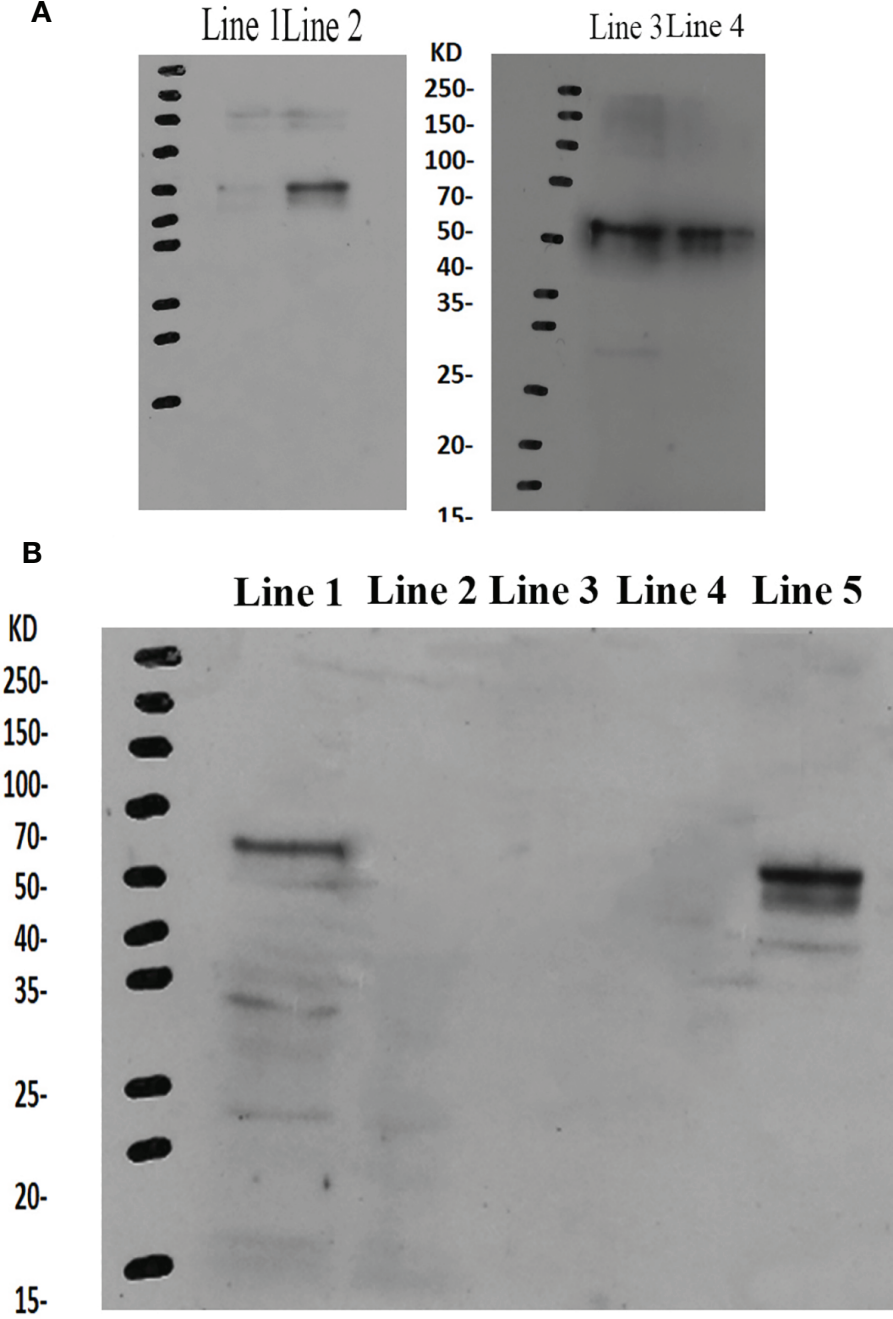
**(A)** Western-blot analysis of *Mn-PDHE1* in the testis and androgenic gland from the reproductive season and non-reproductive season. Line 1: Testis from the non-reproductive season; Line 2: Testis from the reproductive season; Line 3: Androgenic gland from reproductive season; Line 4: Androgenic gland from the non-reproductive season. **(B)** Western-blot analysis of *Mn-PDHE1* in the different ovarian developmental stages. Line 1: Ovarian developmental stage I; Line 2: Ovarian developmental stage II; Line 3: Ovarian developmental stage III; Line 4: Ovarian developmental stage IV; Line 5: Ovarian developmental stage V.

The western blot analysis showed that the molecular mass of *Mn-PDHE1* was approximately 50 kDa, which was slightly larger than that of the predicted molecular weight. Jin et al. (13) previously reported that mRNA expression of *Mn-PDHE1* in the testis was higher during the reproductive season than during the non-reproductive season. Clear protein bands were visible in the testis samples from the reproductive season, but the bands were blurry in the samples from the non-reproductive season. However, clear bands were observed in the androgenic gland for both of the two seasons (**Figure 6A**).

During ovarian development, clear bands were only observed for stages OI and OV, indicating that *Mn-PDHE1* was transcribed during these stages. *Mn-PDHE1* protein expression was more up-regulated in the OV stage than in the OI stage. No bands were observed in stages OII, OIII, and OIV, indicating that *Mn-PDHE1* protein was not transcribed during these periods of development (**Figure 6B**).

### *In Situ* Hybridization Analysis

**Figure 7:**
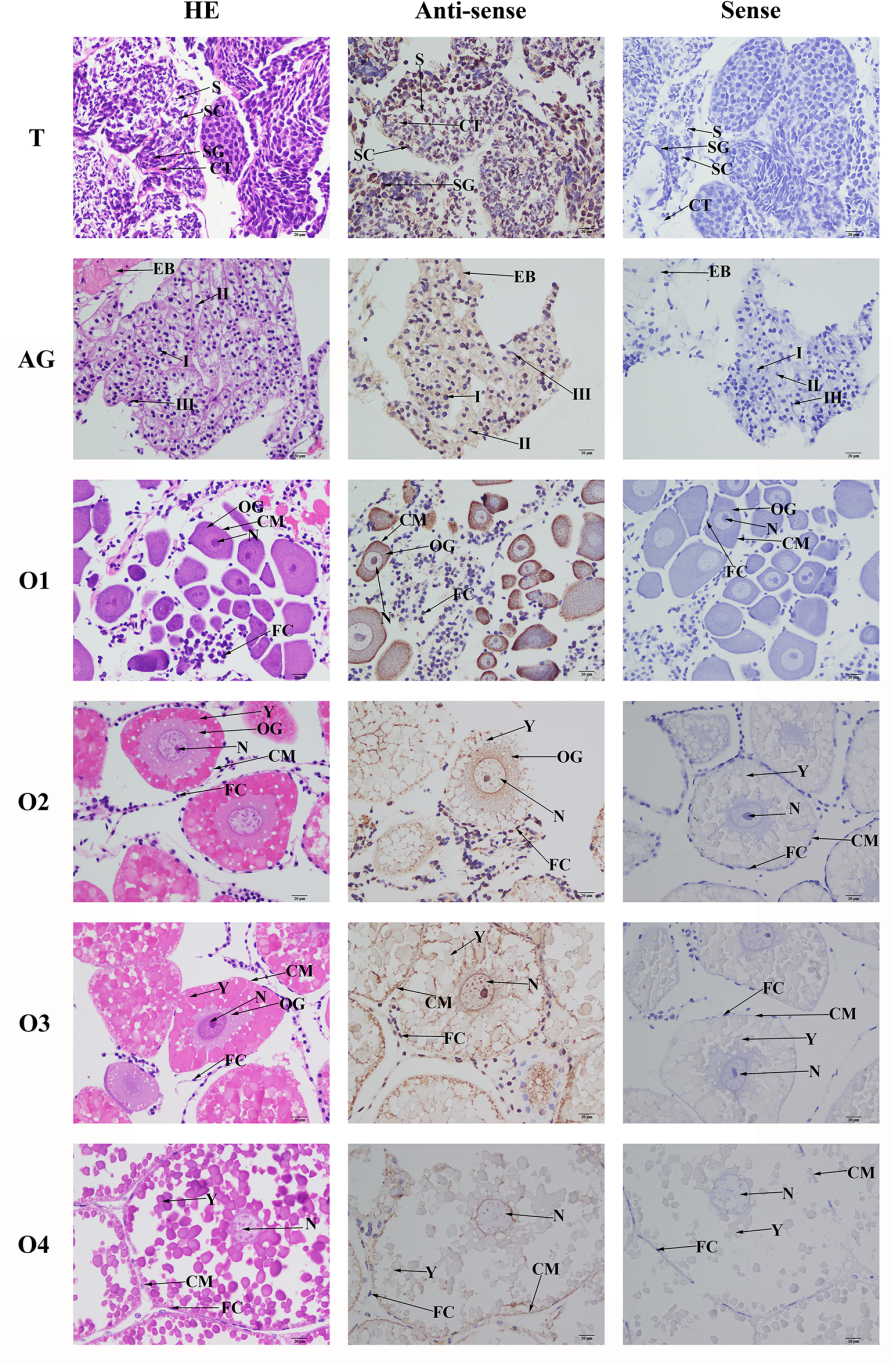
Location of *PDHE1* gene was detected in testis, androgenic gland and ovary of *M*. *nipponense* by using *in situ* hybridization. ST, seminiferous tubule; SG, Spermatogonia; SC, spermatocyte; S, sperm; CT, collecting tissue; E, wall epithelium; EM, eosinophilic matrix; VD, vas deferens; EB, ejaculatory bulb; OG, oogonium; OC, oocyte; CM, cytoplasmic membrane; N, nucleus; Y, yolk granule; FC, follicle membrane. Scale bars = 20 μm.

HE staining showed that the androgenic gland included androgenic gland cells and the ejaculatory bulb. The cells in the testis included spermatogonia, spermatocytes, sperm, and collecting tissues. Strong mRNA signals for *Mn-PDEH1* in the androgenic gland were only observed in the ejaculatory bulb surrounding the androgenic gland cells (**Figure 7**). mRNA signals for *Mn-PDHE1* were only observed in spermatogonia in the testis (**Figure 7**).

Oogonia and follicle cells, which are derived from ovarian epithelial cells, were observed in stage OI. The follicular cavity formed in stage OII, oocyte volume gradually increased in stage OIII, and yolk granules accumulated in the oocytes in stage OIV. mRNA signals for *Mn-PDHE1* were observed in all of the cell types and organelles from stages OI to OIV, including the nucleus, oogonia, oocytes, cytoplasmic membrane, yolk granules, follicle cells, and follicle membrane (**Figure 7**).

### RNAi Analysis

**Figure 8:**
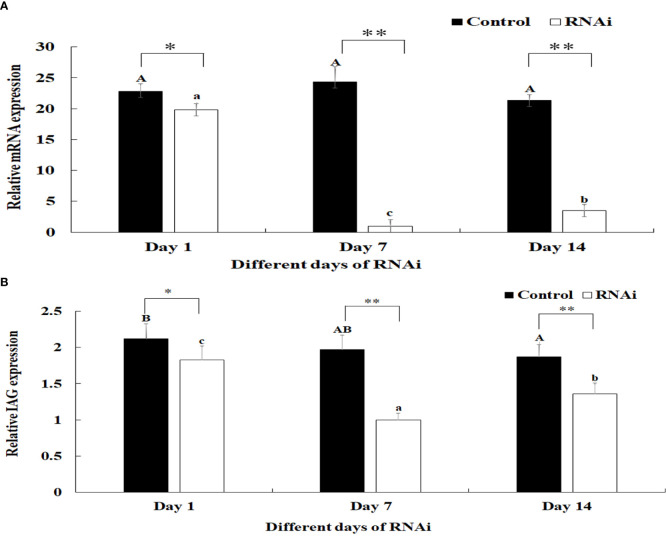
**(A)** Expression characterization of *Mn-PDHE1* at different days after *Mn-PDHE1* dsRNA injection. **(B)** Expression characterization of *Mn-IAG* at different days after *Mn- PDHE1* dsRNA injection. The amount of *Mn-PDHE1* and *Mn-IAG mRNA* was normalized to the *EIF* transcript level. Data are shown as mean ± SD (standard deviation) of tissues from three separate individuals. Capital letters indicated expression difference between different days after vehicle injection in control group. Lowercase indicated expression difference between different days after *Mn-PDHE1 dsRNA* injection in RNAi group. *(p < 0.05) and **(p < 0.01) indicates significant expression difference between the RNAi group and control group at the sample day.

The relative mRNA expression levels of *Mn-PDEH1* were measured in the testis on days 1, 7, and 14 after the *Mn-PDHE1* dsRNA treatment in both the RNAi and control groups. *Mn-PDHE1* mRNA expression remained at a stable level in the control group and did not differ significantly over time (*p* > 0.05). In contrast, *Mn-PDHE1* mRNA expression in the RNAi group gradually decreased from day 1 to 7, reached the lowest level on day 7, and increased slightly by day 14. The *Mn-PDHE1* mRNA expression level was > 95% and 85% lower on days 7 and 14 in the RNAi group, respectively, compared to the control group on the same day (*p* < 0.01) (**Figure 8A**).

Knockdown of the expression of *Mn-PDHE1* also had a positive regulatory effect on the expression of *Mn-IAG*. In the RNAi group, mRNA expression of *Mn-IAG* was > 49% and 31% lower on days 7 and 14, respectively, compared to the control group on the same day (*p* < 0.01) (**Figure 8B**).

**Figure 9:**
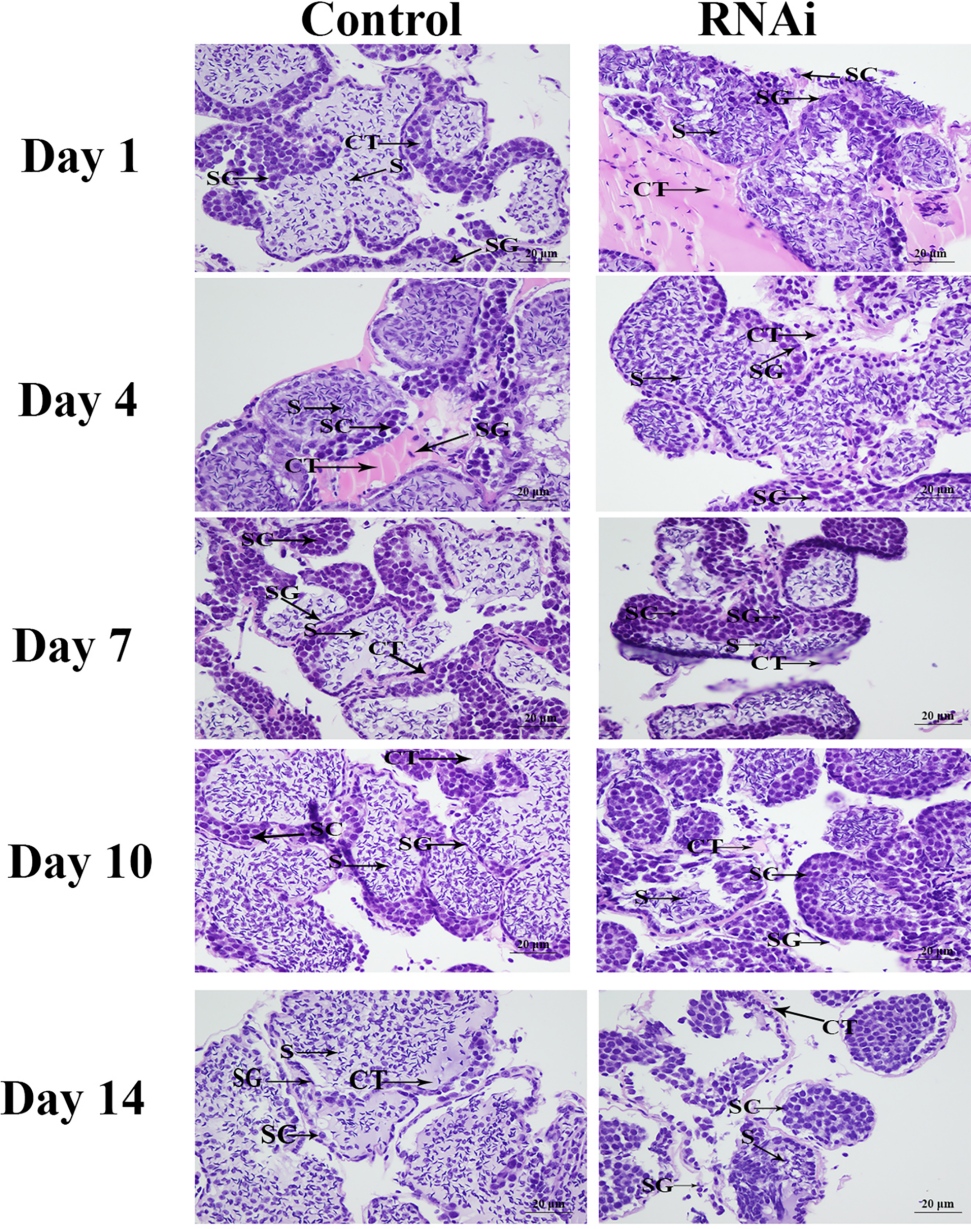
The histological observations of testis between RNAi and control group. SG, Spermatogonia; SC, spermatocyte; S, sperm; CT, collecting tissue. Scale bars = 20 μm.

HE staining showed that sperm (> 60%) were the dominant cells in the testis in the control group, and spermatogonia and spermatocytes were rare. The morphology of the cells did not differ significantly over time. Compared with the control group, the number of sperm in the RNAi group was dramatically lower on day 7. On day 14, sperm accounted for only about 1% of the cells in this group, whereas the numbers of spermatogonia and spermatocytes had increased (**Figure 9**).

## Discussion

The *PDHc* is involved in the glycolysis pathway, catalysing the oxidative decarboxylation of pyruvate to become acetyl-CoA, which is required by the TCA cycle to generate ATP (14, 15). *PDHE1* is the key enzyme component of the *PDHc*, as it promotes ATP generation in the glycolysis/gluconeogenesis and TCA metabolic pathways (16, 17). Jin et al. (13) previously predicted that *PDHE1* plays important roles in generating ATP for male sexual development in *M*. *nipponense*. In this study, we analysed the functions of *PDHE1* in *M*. *nipponense*, focusing especially on the regulatory effects of *Mn-PDHE1* on *Mn-IAG* expression and on testis development. Our results may be useful for developing a technique to regulate testis development in this species. To date, the actual functions of *PDHE1* have not been reported for any crustacean species.

The BLASTP analysis showed 90.72% similarity between *Mn-PDHE1* and *PDHE1* of *P. vannamei*, and *Mn-PDHE1* also shared over 70% sequence identity with *PDHE1* from other insect species. To the best of our knowledge, the only *PDHE1* sequence available for a crustacean species prior to our study was that for *P. vannamei*. The neighbour-joining analysis showed that *Mn-PDEH1* and the *PDHE1* from *P. vannamei* clustered together as a group, and those from the insect species clustered together as another group. Thus, *Mn-PDEH1* has the closest evolutionary relationship with the crustacean species and a much longer evolutionary relationship with insect species, which is consistent with evolutionary analysis of other genes in *M*. *nipponense* (22, 30, 31). More *PDHE1* sequences from other species are needed, especially for crustacean species, in order to better analyse the evolutionary relationship of *Mn-PDHE1*.

The physiological functions of *PDHE1* in *M*. *nipponense* can be reflected by the qPCR results of expression in various adult tissues and developmental stages. For example, qPCR analysis of *PDHE1* in the roundworm *Ascaris suum* revealed that the Type I sequence was highly expressed in adult muscle, whereas the Type II sequence was abundant in the third-stage larvae as well as in adult muscle (32). In *Streptococcus mutans*, Korithoski et al. (33) found that *PDHE1α* expression dramatically increased during adaptation to acidic growth conditions. They also reported that *PDHE1α* expression increased in conditions favouring heterofermentative growth, decreased in the presence of excess glucose, and increased during the stationary phase compared with the mid-log phase of growth. In adult mice, *PDHE1α* showed testis-specific expression as well as somatic forms (34). They detected expression of *PDHE1α* in spermatogonia, Leydig cells, and Sertoli cells at a low level in somatic form. Our *in situ* hybridization analyses showed that *PDHE1α* was abundant in spermatocytes. The highest expression of *Mn-PDHE1* was detected in the testis, which indicated that the activities of the TCA cycle were sufficient to generate ATP and promote male sexual development in *M*. *nipponense*. *Mn-PDHE1* was more highly expressed during the larval developmental stages than during the post-larval developmental stages of juvenile *M*. *nipponense*, and the highest expression was observed in L15, indicating that *PDHE1* promotes the metamorphosis process in *M*. *nipponense* (35, 36). During post-larval development, the highest expression level of *Mn-PDHE1* occurred in PL5, and it gradually decreased to PL15. Previous histological studies showed that both the testis and ovary could be differentiated at PL5, and they matured at PL19 and PL22, respectively (5). Thus, the peak expression in PL5 indicated that *PDHE1* plays essential roles in promoting gonad differentiation. The gender of *M*. *nipponense* can be distinguished for the first time by the naked eye at PL25, and the expression in PL25♂ was 2-fold higher than that in PL25♀. This result was consistent with results of the qPCR analysis of different tissues, which showed that *PDHE1* plays more essential roles in male sexual development in *M*. *nipponense*.

Western blot analysis revealed that the molecular weight of *Mn-PDHE1* protein was about 50 kDa, which was slightly larger than that of predicted molecular weight. This result indicates that the *Mn-PDHE1* protein was modified after transcription. Jin et al. (13) previously reported that *Mn-PDHE1* mRNA was more highly expressed in the testis and androgenic gland during the reproductive season than during the non-reproductive season. Our western blot results showed that *Mn-PDHE1* proteins were transcribed in the testis and androgenic gland during the reproductive season and non-reproductive season, while the protein expression in the testis from non-reproductive season may be quite low. Together these findings indicate that *PDHE1* promotes testis development during the reproductive season. qPCR analysis of the ovarian reproductive cycle revealed that the high expression levels of PDHE1 were observed at the OI and OV stages. Western-blot analysis of the ovarian reproductive cycle indicated that the *Mn-PDHE1* protein was only transcribed in the OI and OV stages, and expression in stage OV was up-regulated relative to that in stage OI, which is consistent with that of qPCR analysis. Oogonia and follicle cells, which were derived from ovarian epithelial cells, were observed in stage OI. In stage OV, the mature oocytes were excreted (24).

*In situ* hybridization and immunostaining analysis showed that *PDHE1α* was abundant in spermatocytes in adult mice (34). We only observed strong mRNA signals of *Mn-PDHE1* in spermatogonia in the testis in *M*. *nipponense*, indicating that *PDHE1* plays essential roles in activating the testis developmental process. In the androgenic gland, strong mRNA signals were only observed in the ejaculatory bulb surrounding the androgenic gland cells. This finding indicated that *PDHE1* was not directly secreted by the androgenic gland cells in *M*. *nipponense* but that it plays essential roles in maintaining the normal structure and function of the androgenic gland (22, 30, 31). In the ovary, *PDHE1* was widely observed in all cell types, indicating that it was involved in the whole ovarian developmental process (24).

RNAi is an efficient method to analyse gene functions (37–39), and it has been widely used in studies of *M*. *nipponense* (24, 40). *PDHE1* was shown to be involved in the glycolysis/gluconeogenesis and TCA metabolic pathways, promoting the ATP generation, which has been proven to play essential roles in testis development in adult mice (34). Jin et al. (13) predicted that *PDHE1* might be involved in male sexual development in *M*. *nipponense*. Our qPCR analysis revealed that injection of *Mn-PDHE1* dsRNA efficiently inhibited the mRNA expression of *Mn-PDHE1* on days 7 and 14 after treatment. To analyse the regulatory roles of *PDHE1* on *IAG*, the expression level of *Mn-IAG* was also measured in the same prawns. The expression of *Mn-IAG* was also significantly decreased on days 7 and 14 after treatment with *Mn-PDHE1* dsRNA, indicating that *PDHE1* positively regulated the expression of *IAG* in *M*. *nipponense*. Previous studies reported that *IAG*, which is secreted by the androgenic gland, is involved in male sexual differentiation and development in many crustacean species (41–43). Knockdown of the expression of *IAG* resulted in sex reversal in *Macrobrachium rosenbergii* (44). The positive regulatory relationship between *PDHE1* and *IAG* highlights the important functions of *PDHE1* in male sexual development in *M*. *nipponense*, as stipulated by Jin et al. (13). Morphologically, the number of sperm was significantly lower beginning on day 7 in the *Mn-PDHE1* dsRNA treatment group compared to the control group. On day 14, sperm accounted for only 1% of the cells in the RNAi group, which indicated that *Mn-PDHE1* positively regulates testis development in *M*. *nipponense*.

In conclusion, our results indicate that *PDHE1* is involved in the metamorphosis and gonad development of *M*. *nipponense*, and it is especially important for male sexual development. *Mn-PDHE1* was shown to positively regulate the expression of *IAG* and testis development in *M. nipponense*. This study provides essential data needed to establish a technique to regulate testis development in *M*. *nipponense*.

## Data Availability Statement

The original contributions presented in the study are included in the article/supplementary material. Further inquiries can be directed to the corresponding author.

## Ethics Statement

The animal study was reviewed and approved by Freshwater Fisheries Research Center, Chinese Academy of Fishery Sciences.

## Author Contributions

SJin designed and wrote the manuscript. YH performed the RNAi analysis. HF supervised the experiment. SJia and YX provided the experimental prawns. HQ performed the qPCR analysis. WZ performed the western-blot analysis. YG performed the *in situ* hybridization analysis. YW performed the histological observations. All authors contributed to the article and approved the submitted version.

## Funding

This research was supported by grants from the National Key R&D Program of China (2018YFD0900201); Central Public-interest Scientific Institution Basal Research Fund CAFS (2021JBFM02; 2020TD36); Jiangsu Agricultural Industry Technology System (JATS[2020]461); the China Agriculture Research System-48 (CARS-48); the New cultivar breeding Major Project of Jiangsu province (PZCZ201745).

## Conflict of Interest

The authors declare that the research was conducted in the absence of any commercial or financial relationships that could be construed as a potential conflict of interest.

## Publisher’s Note

All claims expressed in this article are solely those of the authors and do not necessarily represent those of their affiliated organizations, or those of the publisher, the editors and the reviewers. Any product that may be evaluated in this article, or claim that may be made by its manufacturer, is not guaranteed or endorsed by the publisher.
